# The impact of wearable resistance training on strength, speed, and agility: a systematic review and meta-analysis

**DOI:** 10.7717/peerj.20519

**Published:** 2026-01-02

**Authors:** Shuairan Li, Qiwei Wang, Yingying Cao, Xiaoli Huang, Yuanyuan Luo, Jing Mi

**Affiliations:** 1 International Joint Laboratory on High Performance Sports Research, Beijing Sport University, Beijing, China; 2 Sports Coaching College, Beijing Sport University, Beijing, China; 3 Moray House School of Education and Sport, The University of Edinburgh, Edinburgh, United Kingdom; 4 School of Sports, Xian University, Xian, Shanxi, China; 5 Dazhou Vocational College of Traditional Chinese Medicine, Dazhou, Sichuan, China

**Keywords:** Wearable resistance training, Strength, Speed, Agility, Performance

## Abstract

**Objective:**

To conduct a systematic assessment of the impact of wearable resistance training (WRT) on muscular strength, speed, and agility, while examining the influence of critical training parameters as moderating factors.

**Methods:**

A systematic review was undertaken to identify eligible studies. Literature searches were conducted in PubMed, Web of Science, EBSCO databases from inception to October 31st, 2025. This study employed the Cochrane Collaboration tool built into Review Manager 5.4 for literature quality assessment, and utilized Stata version 18.0 for meta-analysis, including pooled effect size calculation, subgroup analysis, and publication bias assessment.

**Results:**

The Meta-analysis encompassed 19 studies with a total of 233 participants. It is revealed that WRT produced significant improvements in jumping power (standardized mean difference (SMD) = −0.60; 95% CI [−1.07 to −0.14]; *p* = 0.01), and agility performance (SMD = −0.44, 95% CI [−0.58 to −0.30], *p* < 0.001). Subgroup analyses demonstrated that the most pronounced jumping power enhancements occurred with training frequencies of three sessions per week (SMD = −0.47, 95% CI [−0.92 to −0.02], *p* = 0.038). Additionally, external loading protocols utilizing 8–19% of body weight yielded superior improvements (SMD = −0.17, 95% CI [−0.37 to 0.03], *p* = 0.032). Finally, subgroup analyses revealed no significant moderating effects for wearable device placement, participant age, or study population characteristics (*p* > 0.05).

**Conclusion:**

WRT effectively enhances jumping power and agility. Although it contributes to some improvement in speed and maximal strength, the effect is not statistically significant. Further high-quality studies are needed in the future to validate these findings.

## Introduction

Short-distance sprinting, agility, and explosive jumping are widely recognized as critical components of athletic performance (Macadam, Simperingham & Cronin, 2019). Although conventional resistance training methods, like dumbbell and barbell exercises, are effective for building muscular strength, their direct application to sport-specific performance is still a matter of contention. Consequently, enhancing strength in alignment with sport-specific movement patterns has become a key goal for coaches and practitioners (Cleary Dolcetti et al., 2019).

Wearable resistance training (WRT) has gained attention as a promising strategy for providing sport-specific overload in dynamic and functional contexts. Unlike conventional resistance methods, WRT enables external loading without disrupting natural movement patterns, thus integrating strength development seamlessly into sport-specific activities (Cleary Dolcetti et al., 2019; Promsri et al., 2024). By directly applying resistance to the body, WRT can enhance neuromuscular recruitment speed and provide an ideal training stimulus tailored to the demands of high-intensity athletic actions. The physiological mechanisms underlying WRT effectiveness include enhanced motor unit recruitment, improved rate of force development, and optimized neuromuscular coordination, while biomechanically, WRT maintains sport-specific movement kinematics and kinetic chain activation patterns that facilitate superior transfer to competitive performance (Macadam, Cronin & Simperingham, 2017).

Previous research has indicated that WRT can have a positive effect on strength (assessed through maximal strength tests such as 1RM squat and bench press, and explosive power tests including countermovement jump), speed (evaluated *via* short-distance sprint protocols), and agility (measured using tests such as the 505 agility test), potentially enabling a more efficient transfer of training adaptations to competitive performance (Clark et al., 2010; Alcaraz et al., 2008; Feser et al., 2023; Simperingham & Cronin, 2014). However, it is still uncertain which specific physical attribute benefits most from WRT, and the mechanisms underlying its sport-specific transfer and performance enhancement are not fully comprehended. For practitioners, understanding the advantages and limitations of WRT regarding training prescription and targeted adaptations is of significant value.

Therefore, this study aims to examine the long-term effects of WRT on athletic performance by systematically evaluating its impact on strength, speed, and agility. Subgroup analyses are performed based on critical training variables, including external load, training frequency, and intervention duration. This comprehensive meta-analysis represents the first systematic attempt to quantify WRT’s differential effects across multiple performance domains while identifying optimal training parameters, thereby providing evidence-based guidelines for practitioners seeking to maximize sport-specific training adaptations.

## Methods

This review adhered strictly to the Preferred Reporting Items for Systematic reviews and Meta-Analyses (PRISMA) guidelines, Page et al. (2021) with all necessary components reported as outlined (see Supplementary Checklist: https://www.crd.york.ac.uk/PROSPERO/). The review protocol was registered in advance in the PROSPERO database (Registration ID: CRD420250655386; registration date: February 27, 2025).

### Literature search strategy and screening process

A comprehensive literature search was systematically conducted across Web of Science, PubMed, and EBSCO databases, covering all records from their inception up to October 31^th^, 2025. The review was carried out in strict adherence to the PRISMA guidelines (Page et al., 2021). To ensure comprehensive coverage, domain experts utilized the Medical Subject Headings (MeSH) database to identify pertinent keywords and synonyms. Search terms included, but were not restricted to: “resistance training,” “weight training,” “strength training,” “weight-bearing exercise,” “wearable resistance,” “loaded movement training,” “muscle strength,” “power,” “force production,” “athletic performance,” “speed,” “velocity,” “sprint performance,” “running speed,” “physical functional performance,” “agility,” “change of direction ability,” “quickness,” and “motor agility.” A comprehensive description of the search strategy is available in Supplemental Material S1.

### Study selection and eligibility criteria

The inclusion criteria for this review were set based on the PICOS framework, with the main elements specified as follows: Population (P): Healthy individuals; Intervention (I): Strength, sprint, or change-of-direction training utilizing wearable resistance devices (*e.g*., weighted vests); Comparison (C): Comparable training protocols performed without wearable resistance devices; Outcomes (O): Primary outcomes included strength, speed, and agility, assessed through quantitative performance metrics; Study Design (S): Only randomized controlled trials (RCTs) were considered to ensure solid causal inference. Cross-sectional studies were omitted.

Inclusion criteria: (1) The study population consisted of healthy athletes from diverse sports disciplines. (2) The intervention included the application of wearable resistance devices. (3) Outcomes were assessed using the following performance-based measures: Strength: squat jump, vertical jump, one-repetition maximum (1RM) back squat, bench press, and power clean; Speed: short-distance sprint performance and ground contact time; Agility: shuttle run, 505 test, figure-8 run, and T-test. (4) The study was designed as a randomized controlled trial (RCT). (5) The publication was in English. Wearable resistance devices were additionally classified according to manufacturer specifications (see Supplement Material). Exclusion criteria: (1) Studies involving disabled athletes, injured individuals, or special populations in rehabilitation. (2) Interventions that included extra elements beyond wearable resistance (*e.g*., conventional strength training, perceptual-cognitive training, nutritional or pharmacological supplements). (3) Intervention with the load positioned on the upper limbs. (4) Studies with redundant content or overlapping datasets. (5) Studies for which the full text could not be obtained. (6) Low-quality studies characterized by insufficient experimental control or defective statistical analysis. (7) Studies restricted to acute, single-session interventions.

### Data extraction and quality assessment

For this meta-analysis, data screening and extraction were carried out independently by two researchers (Li and Luo). Any discrepancies were settled through discussion with a third researcher (Cao). A predefined data extraction form was employed to systematically document and categorize all relevant study information. The extracted data included: (1) Basic study characteristics: author names, country, and publication year; (2) Participant characteristics: sample size, age, sex, training background, competitive experience, and performance level; (3) Intervention details: study design, intervention duration, training frequency, loading location, load magnitude and type, as well as the classification of outcome measures for strength, speed, and agility; (4) Outcome variables: performance indicators related to strength, speed, and agility, presented as means and standard deviations; (5) Multiple outcomes within studies: if a single study reported multiple outcomes in any performance domain (strength, speed, or agility), each was considered as a separate analytical entry; (6) Load categorization: load intensity was classified as light, moderate, or heavy based on body weight percentage; (7) Wearable resistance protocol: information on when the wearable resistance was applied (*e.g*., during warm-up, main session, or cooldown). For studies with incomplete or missing data, the corresponding author was contacted to obtain the required information. If no response was received within 48 h, a follow-up email was dispatched. Studies that did not respond were omitted from the final analysis.

The risk of bias was evaluated using a modified version of the Cochrane Collaboration’s Risk of Bias Assessment Tool (Higgins et al., 2011), which evaluates seven key methodological domains: (1) random sequence generation; (2) allocation concealment; (3) blinding of participants and personnel; (4) blinding of outcome assessment; (5) completeness of outcome data; (6) selective reporting of results; and (7) other potential sources of bias. A study was deemed to have a low overall risk of bias if it was assessed as “low risk” across all domains. If one or two domains were rated as “high risk” or “unclear risk”, the study was considered to have a moderate overall risk of bias. Studies with more than two domains judged as “high risk” or “unclear risk” were considered to have a high overall risk of bias.

### Statistical methods

Statistical analyses were performed using Stata version 18.0, encompassing sensitivity analyses, effect size synthesis, meta-regression, subgroup analyses, and evaluation of publication bias. Outcome measures were extracted as mean ± standard deviation (mean ± SD), with corresponding 95% confidence intervals (CIs). Statistical significance was determined at *p* < 0.05 and *p* < 0.01, following the rationale outlined by Andrade (2019), who emphasized that *p*-values should be interpreted in conjunction with effect sizes and confidence intervals to avoid misrepresentation of results. Heterogeneity among studies was assessed using Cochran’s Q test and the I²statistic. If the Q test resulted in *p* > 0.1 and I^2^ < 50%, heterogeneity was considered low, and a fixed-effects model was employed. In cases of substantial heterogeneity, a random-effects model was used (Cheung & Cheung, 2016). Considering the typically small sample sizes in wearable resistance training studies, effect sizes were calculated using Hedges’ guidelines (Kallogjeri & Piccirillo, 2023). Effect sizes were categorized based on the following thresholds: small (SMD < 0.2), moderate (0.2 ≤ SMD < 0.5), large (0.5 ≤ SMD < 0.8), and very large (SMD ≥ 0.8) (Makaruk et al., 2024). A small-sample correction factor was applied following Hedges’ guidelines to reduce estimation bias. In instances of high heterogeneity, sensitivity analyses, meta-regression, and subgroup analyses were conducted to explore potential sources of variation. Publication bias was assessed using funnel plot asymmetry and statistically tested using Begg’s and Egger’s tests (Lin & Chu, 2018). For outcomes with fewer than 10 studies, the trim-and-fill method was applied to address potential publication bias (Sera et al., 2019). Furthermore, given the prevalent effect size dependency commonly observed in wearable resistance training (WRT) studies, a fixed-effects inverse variance weighting method was employed to initially pool similar effect sizes within individual studies prior to conducting the main meta-analysis, with the resulting estimates subsequently incorporated into the overall pooled analysis (Borenstein & Higgins, 2013).

## Results

### Literature search results

**Figure 1:**
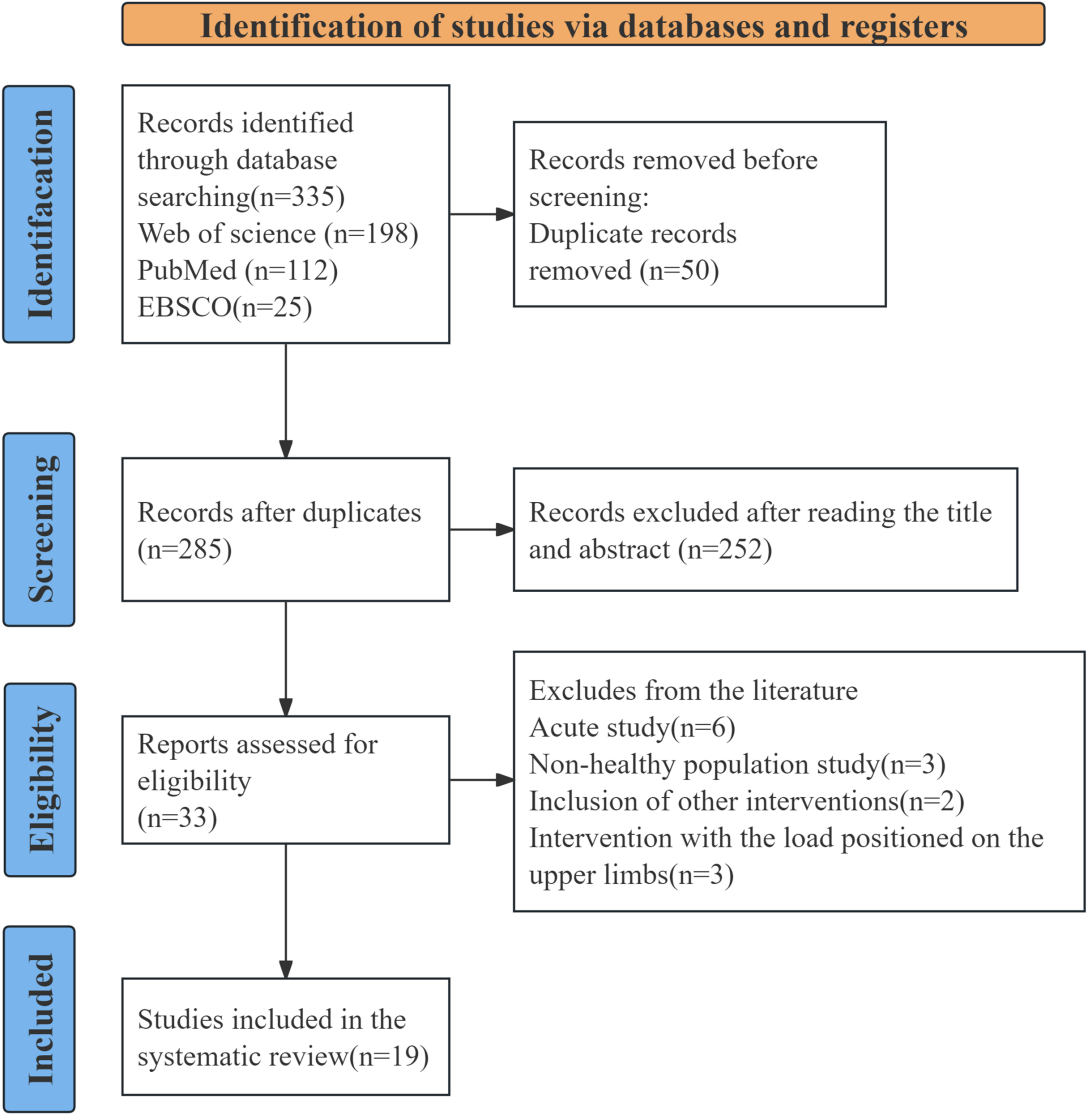
Literature search and screening process.

A comprehensive search of the Web of Science, PubMed, and EBSCO databases identified 335 potentially relevant studies. All citations were imported into EndNote X7, and after removing duplicates, 285 unique records remained. Title and abstract screening led to the selection of 33 articles for full-text evaluation review. Following the application of predefined eligibility criteria, 19 quantitative studies were included in the final meta-analysis (Fig. 1).

### Study characteristics

This meta-analysis included 19 randomized controlled trial (RCT) studies involving 233 participants, comprising 41 adolescents and 192 adults (34 females and 199 males). Wearable resistance loads ranged from 1% to 30% of body weight, primarily positioned on the trunk and lower limbs. Intervention durations varied from 8 days to 10 weeks, with training frequencies ranging from 2 to 7 sessions per week. The outcome measures assessed strength, speed, and agility performance indicators.

### Quality assessment

**Figure 2:**
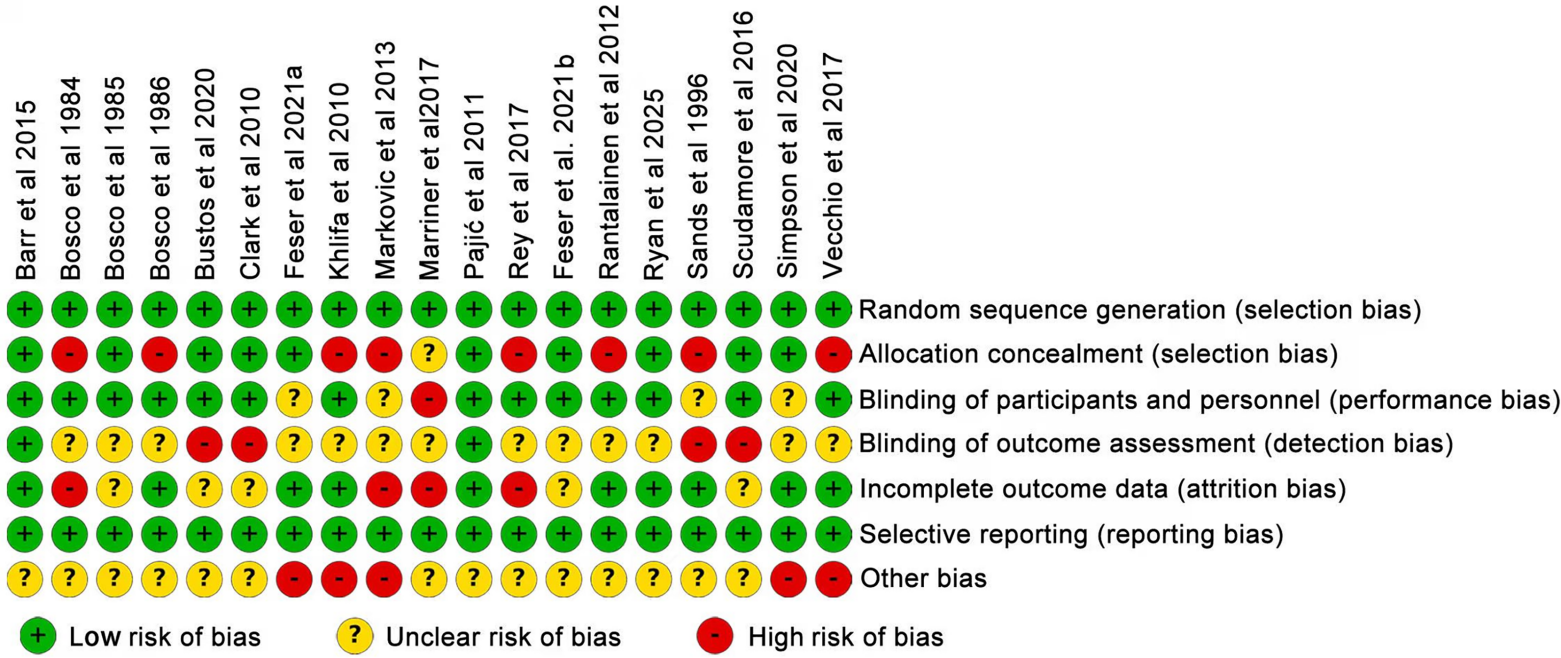
Incorporation of research quality evaluation (Barr et al., 2015; Bosco et al., 1984; Bosco, 1985; Bosco, Rusko & Hirvonen, 1986; Bustos et al., 2020; Clark et al., 2010; Feser et al., 2021a; Khlifa et al., 2010; Markovic et al., 2013; Marriner et al., 2017; Pajić et al., 2011; Rey et al., 2017; Feser et al., 2021b; Rantalainen, Ruotsalainen & Virmavirta, 2012; Ryan et al., 2025; Sands et al., 1996; Scudamore et al., 2016; Simpson et al., 2020; Del Vecchio et al., 2017).

As illustrated in Fig. 2, the risk of bias across all 19 included studies was assessed using a modified Cochrane Collaboration tool. Of these, six studies were deemed to have a low risk of bias across all assessment domains, indicating robust methodological quality. Eight studies demonstrated a high or unclear risk of bias in one or two domains, suggesting a moderate overall risk that could impact the reliability of certain findings. Conversely, five studies were rated as having a high or unclear risk in three or more domains, indicating a potentially significant risk of systematic bias. Therefore, caution should be exercised when interpreting the results derived from these studies.

### Meta-analysis results

#### Sensitivity analysis of the effects of wearable resistance training on strength and speed

Sensitivity analysis represents a fundamental robustness assessment in systematic reviews and meta-analyses. This approach systematically evaluates result stability by modifying analytical decisions or sequentially excluding individual studies to determine whether pooled effect estimates undergo meaningful changes. Results demonstrating consistent effect directions and maintained statistical significance across various scenarios or following the exclusion of any single study are considered robust to individual study influences and methodological assumptions, thereby enhancing confidence in the findings. The present study employed the “leave-one-out” method, a well-established sensitivity analysis strategy that systematically omits each effect size (or study) sequentially, recalculates the pooled effect estimate with its 95% confidence interval (95% CI), and visualizes these iterations in a sensitivity analysis plot.

**Figure 3:**
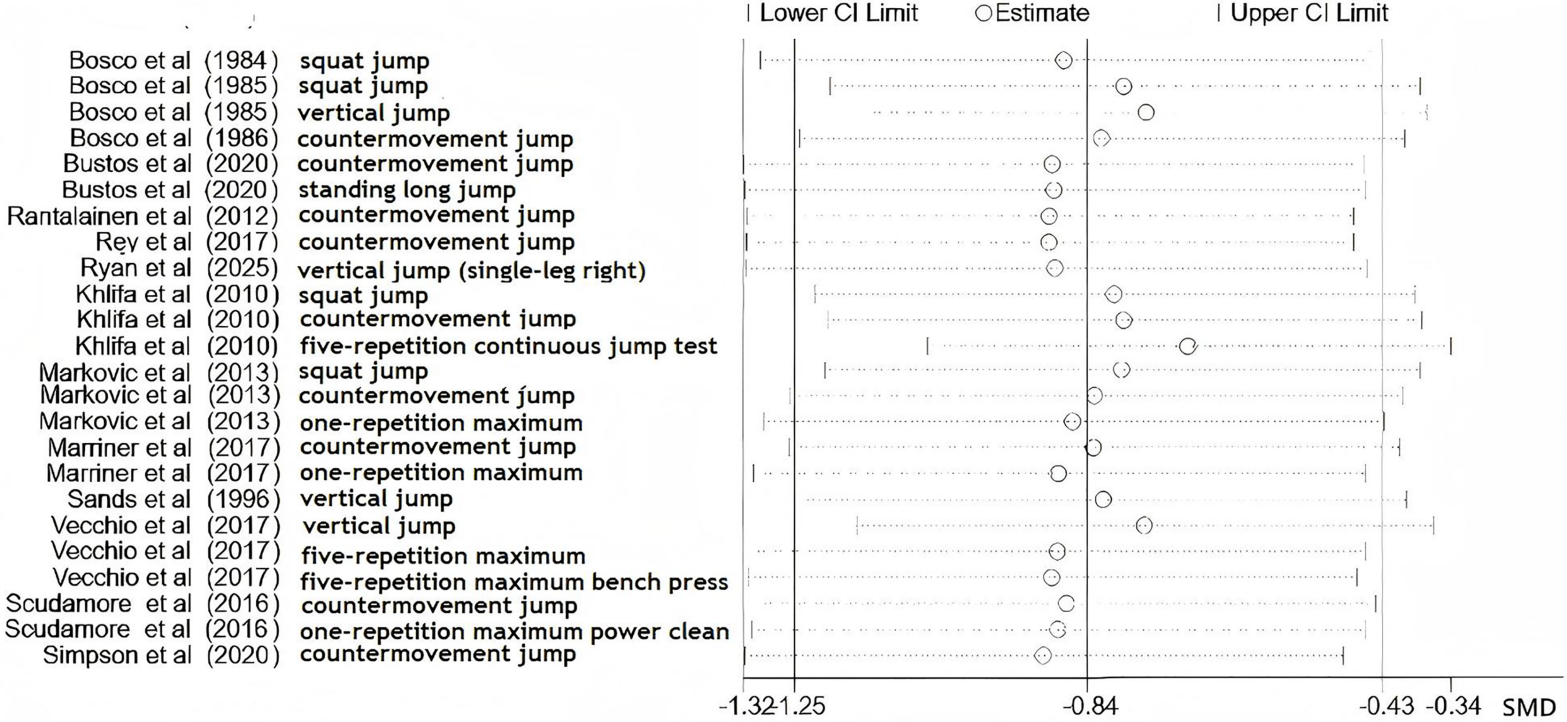
Sensitivity analysis of the effect of wearable resistance training on strength. Records represent outcome-level entries rather than study counts. Outcome codes are defined as follows: a = squat jump (SJ); b = vertical jump (VJ); c = countermovement jump (CMJ); d = one-repetition maximum (1RM) power clean; e = standing long jump; f = five-repetition continuous jump test; g = one-repetition maximum (1RM) squat; h = five-repetition maximum (5RM) squat; i = five-repetition maximum (5RM) bench press (Bosco et al., 1984; Bosco, 1985; Bosco, Rusko & Hirvonen, 1986; Bustos et al., 2020; Khlifa et al., 2010; Markovic et al., 2013; Marriner et al., 2017; Pajić et al., 2011; Rey et al., 2017; Rantalainen, Ruotsalainen & Virmavirta, 2012; Ryan et al., 2025; Sands et al., 1996; Scudamore et al., 2016; Simpson et al., 2020; Del Vecchio et al., 2017).

**Figure 4:**
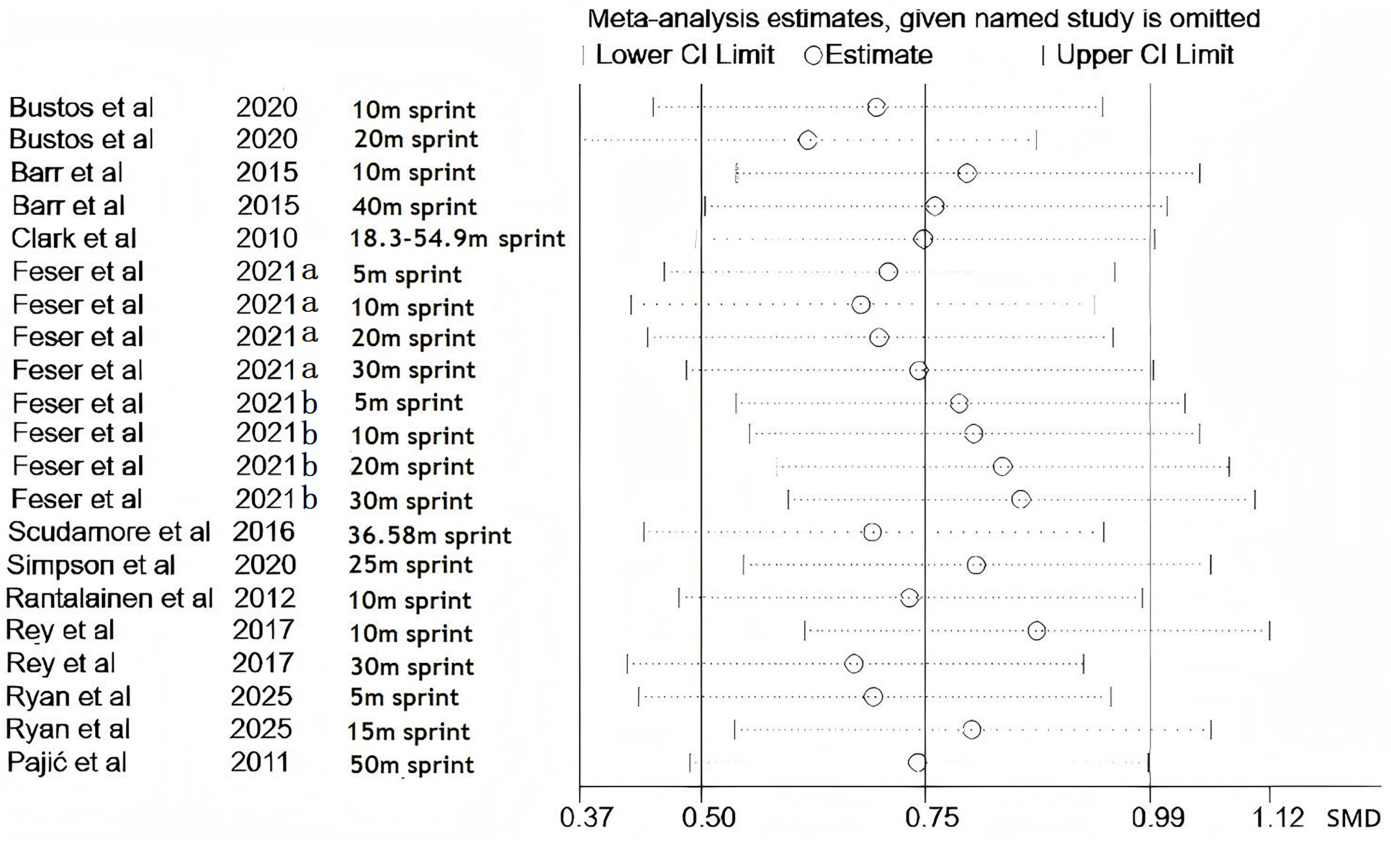
Sensitivity analysis of the effect of wearable resistance training on speed. Records represent outcome-level entries rather than study counts. Outcome codes are defined as follows: a = 10 m sprint; b = 20 m sprint; c = 40 m sprint; d = 18.3–54.9 m sprint; e = 5 m sprint; f = 30 m sprint; g = 36.58 m sprint; h = 25 m sprint; i = 15 m sprint; j = 50 m sprint (Barr et al., 2015; Bustos et al., 2020; Clark et al., 2010; Feser et al., 2021a; Pajić et al., 2011; Rey et al., 2017; Feser et al., 2021b; Rantalainen, Ruotsalainen & Virmavirta, 2012; Ryan et al., 2025; Scudamore et al., 2016; Simpson et al., 2020).

Figure 3 displays the leave-one-out sensitivity analysis for wearable resistance training (WRT) effects on strength parameters. Each horizontal line represents the recalculated pooled standardized mean difference (SMD) with corresponding 95% CI following individual study exclusion. Figure 4 presents parallel sensitivity analyses for velocity parameters. All effect estimates consistently appear right of the null effect line, with 95% CIs excluding zero throughout all iterations.

#### Pooled effects of wearable resistance training on strength, speed, and agility

**Figure 5:**
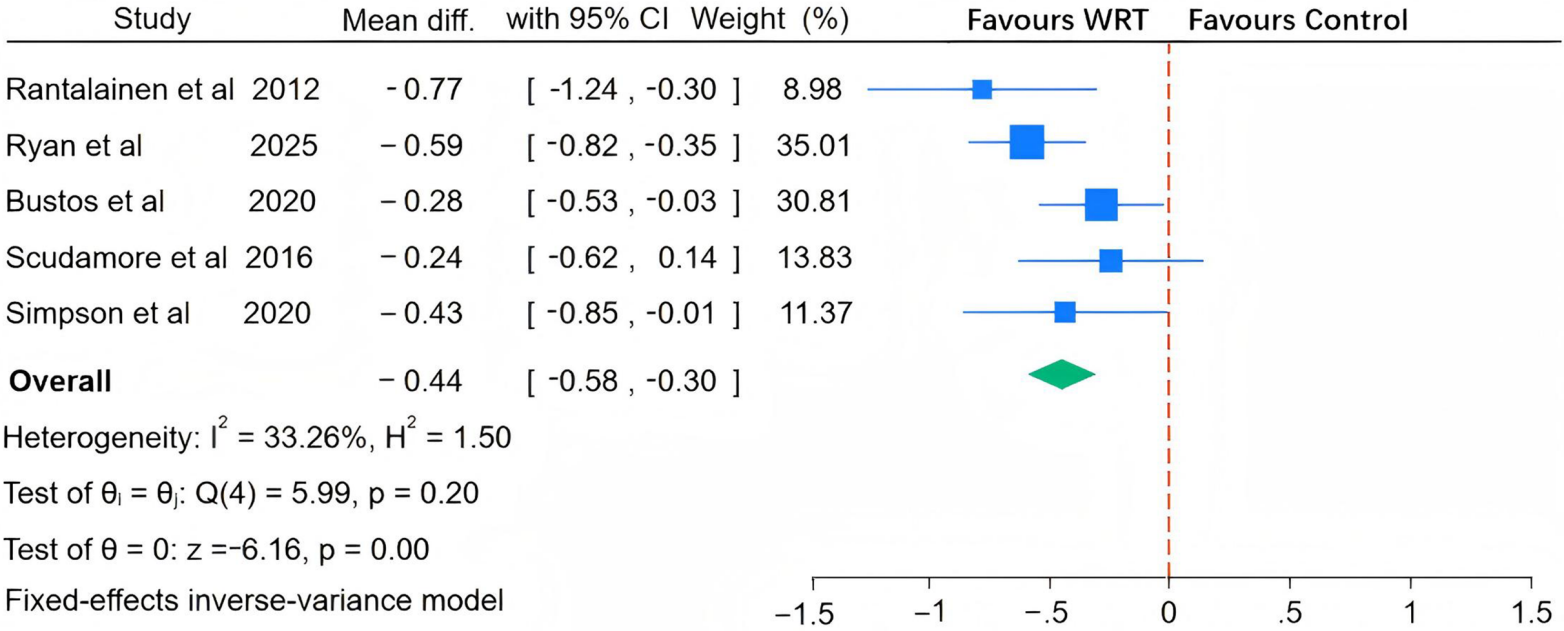
Pooled effect of wearable resistance training on agility performance. Rantalainen: Figure-of-8 run; Ryan: 5-0-5 test, 180° change-of-direction time; Bustos: 20 m shuttle run; Scudamore: 274.3 m long-distance shuttle run, 137.2 m shuttle run time; Simpson: T-test (Bustos et al., 2020; Rantalainen, Ruotsalainen & Virmavirta, 2012; Ryan et al., 2025; Scudamore et al., 2016; Simpson et al., 2020).

**Figure 6:**
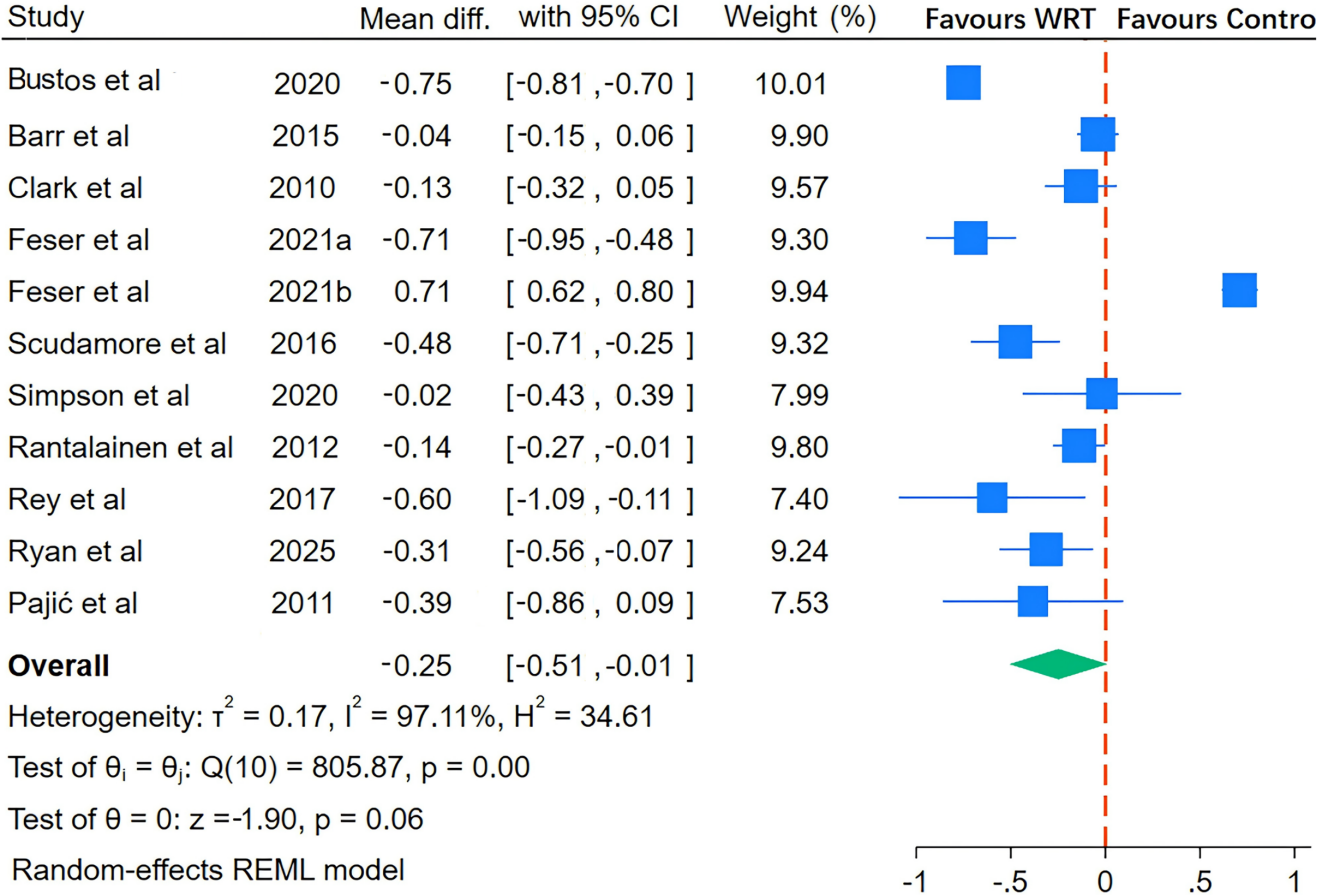
Pooled effect of wearable resistance training on speed performance. Bustos: 10 m sprint, 20 m sprint; Barr: 10 m sprint, 40 m sprint; Clark: 18.3–54.9 m sprint; Feser a: 10 m sprint, 20 m sprint, 5 m sprint, 30 m sprint; Feser b: 10 m sprint, 20 m sprint, 5 m sprint, 30 m sprint; Scudamore: 36.58 m sprint; Simpson: 25 m sprint; Rantalainen: 10 m sprint; Rey: 10 m sprint, 20 m sprint; Ryan: 10 m sprint, 30 m sprint; Ryan: 10 m sprint, 30 m sprint; Pajić: 50 m sprint (Barr et al., 2015; Bustos et al., 2020; Clark et al., 2010; Feser et al., 2021a; Khlifa et al., 2010; Pajić et al., 2011; Rey et al., 2017; Feser et al., 2021b; Rantalainen, Ruotsalainen & Virmavirta, 2012; Ryan et al., 2025; Scudamore et al., 2016; Simpson et al., 2020).

**Figure 7:**
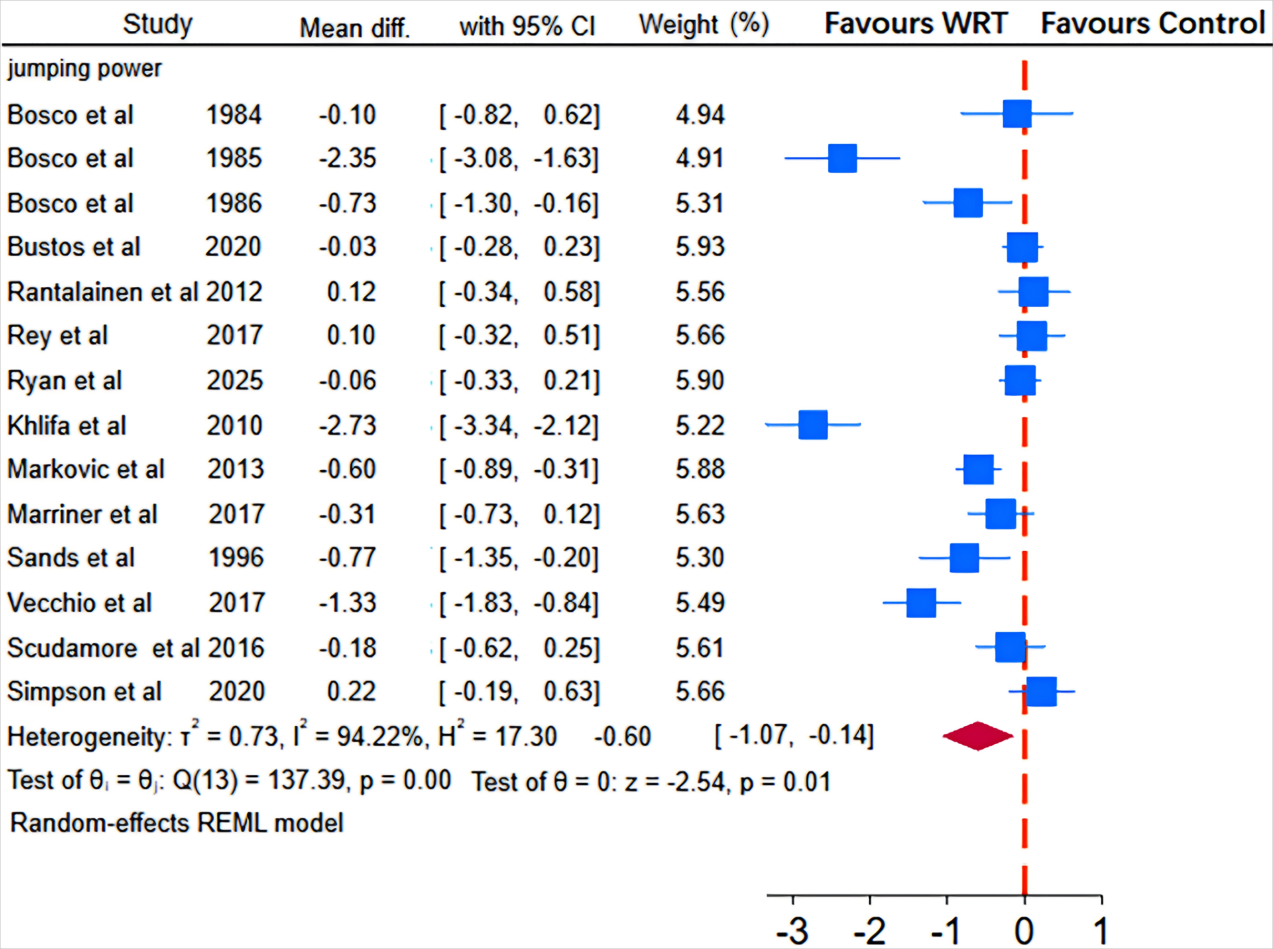
Pooled effect of wearable resistance training on strength performance (Explosive Strength). Bosco 1984: squat jump; Bosco 1985: squat jump, vertical jump; Bosco 1986: squat jump, vertical jump; Bustos: countermovement jump, standing long jump; Rantalainen: countermovement jump; Rey: countermovement jump; Ryan: vertical jump; Khlifa: squat jump, countermovement jump, five-repetition continuous jump test; Markovic: squat jump, countermovement jump; Marriner: countermovement jump; Sands: vertical jump; Vecchio: vertical jump; Scudamore: countermovement jump; Simpson: countermovement jump (Bosco et al., 1984; Bosco, 1985; Bosco, Rusko & Hirvonen, 1986; Bustos et al., 2020; Khlifa et al., 2010; Markovic et al., 2013; Marriner et al., 2017; Rey et al., 2017; Rantalainen, Ruotsalainen & Virmavirta, 2012; Ryan et al., 2025; Sands et al., 1996; Scudamore et al., 2016; Simpson et al., 2020; Del Vecchio et al., 2017).

**Figure 8:**
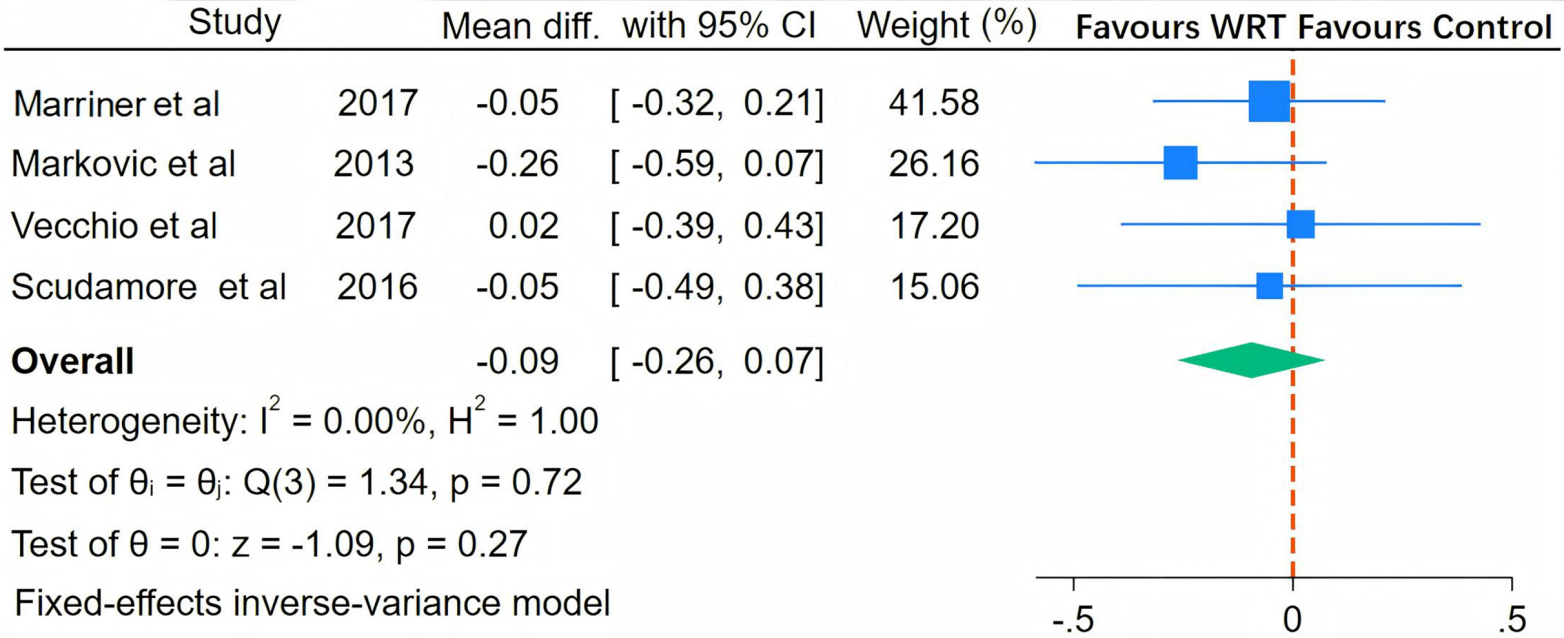
Pooled effect of wearable resistance training on strength performance (Maximal Strength). Marriner: one-repetition maximum (1RM) power clean; Markovic: one-repetition maximum; Vecchio: five-repetition maximum (5RM) squat, five-repetition maximum (5RM) bench press; Scudamore: one-repetition maximum (1RM) power clean (Markovic et al., 2013; Marriner et al., 2017; Scudamore et al., 2016; Del Vecchio et al., 2017).

As illustrated in Figs. 5, 6, 7 and 8, the I^2^ statistics across outcome measures revealed substantial variation, reflecting differing levels of between-study heterogeneity. Specifically, jumping power and speed both exhibited high heterogeneity, indicating significant between-study variability. Agility demonstrated moderate heterogeneity, while maximal strength showed no heterogeneity, suggesting consistent results across studies for this outcome. The Q-statistic tests corroborated these findings: jumping power (Q(13) = 137.39, *p* = 0.01) and speed (Q(10) = 805.87, *p* < 0.001) demonstrated statistically significant heterogeneity, whereas maximal strength (Q(3) = 1.34, *p* = 0.27) and agility (Q(4) = 5.99, *p* < 0.001) exhibited lower or marginally significant heterogeneity levels. Based on these heterogeneity profiles, appropriate analytical models were selected: random-effects models for jumping power and speed, and fixed-effects models for maximal strength and agility to optimize the robustness of pooled estimates.

#### Subgroup analysis of the effects of wearable resistance training on jumping power

Regarding intervention duration, the 1–3 week group did not achieve statistical significance (SMD = −0.11, *p* = 0.309), whereas the 6–8 week group exhibited a positive trend (SMD = 0.13, *p* = 0.114). The between-group difference approached significance (*p* = 0.08). In terms of training frequency, the 3 sessions/week group demonstrated optimal effects (SMD = −0.47, *p* = 0.038), while the 7 sessions/week group paradoxically exhibited negative effects (SMD = 0.22, *p* = 0.041). The between-group difference was significant (*p* = 0.04). Concerning resistance load, training with 8–19% body weight load yielded significant effects (SMD = −0.17, *p* = 0.032), significantly outperforming the low-load group (SMD = 0.18, *p* = 0.099), with a statistically significant between-group difference (*p* = 0.01). For other variables, including placement location, age, and participant population type, subgroup analyses revealed no significant moderating effects (all between-group *p*-values > 0.1). However, it should be noted that these analyses may be constrained by insufficient sample sizes and between-study heterogeneity, warranting further validation through larger-scale future research.

### Publication bias assessment

**Figure 9:**
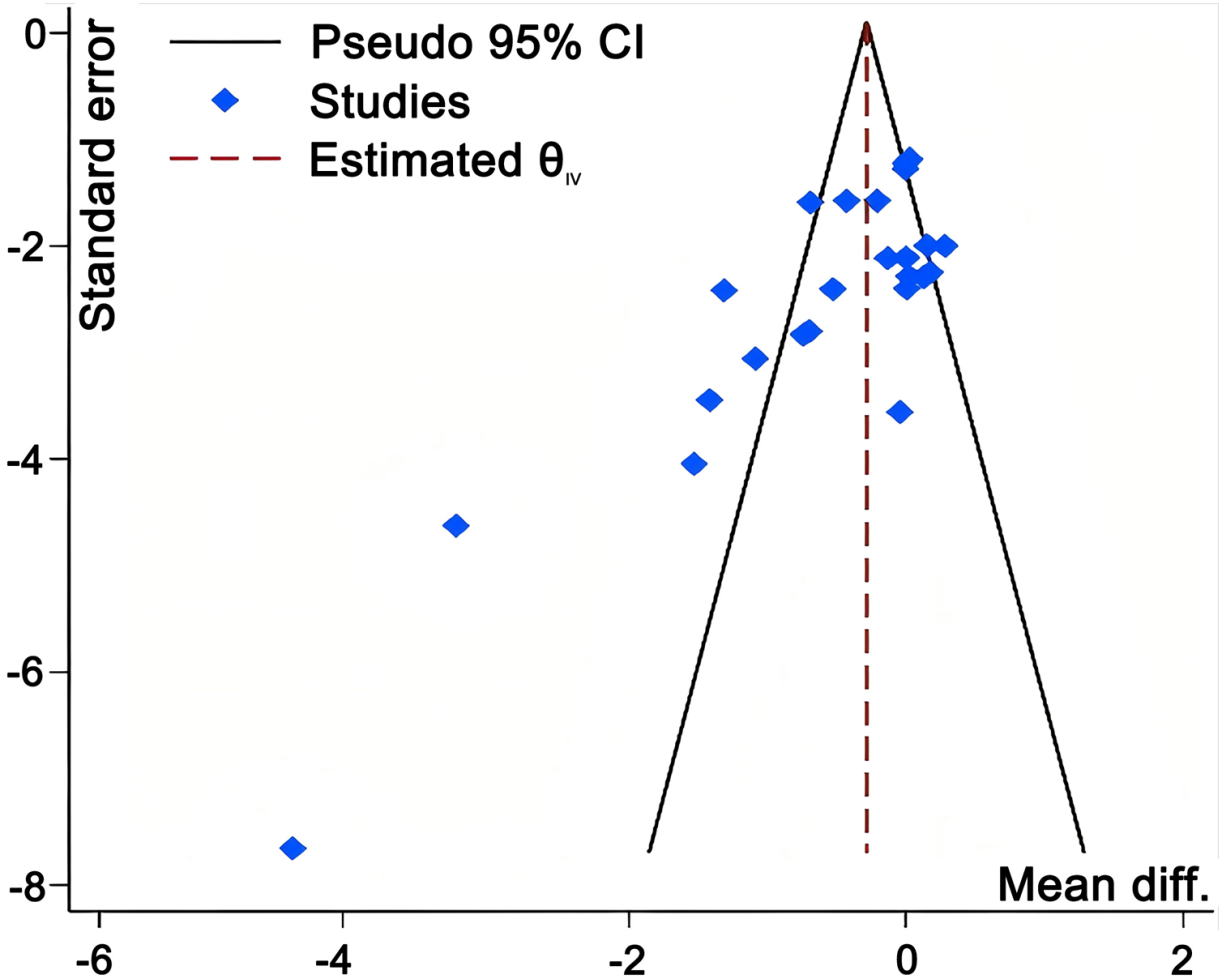
Funnel plot evaluating potential publication bias in studies investigating the effects of WRT on strength outcomes.

**Figure 10:**
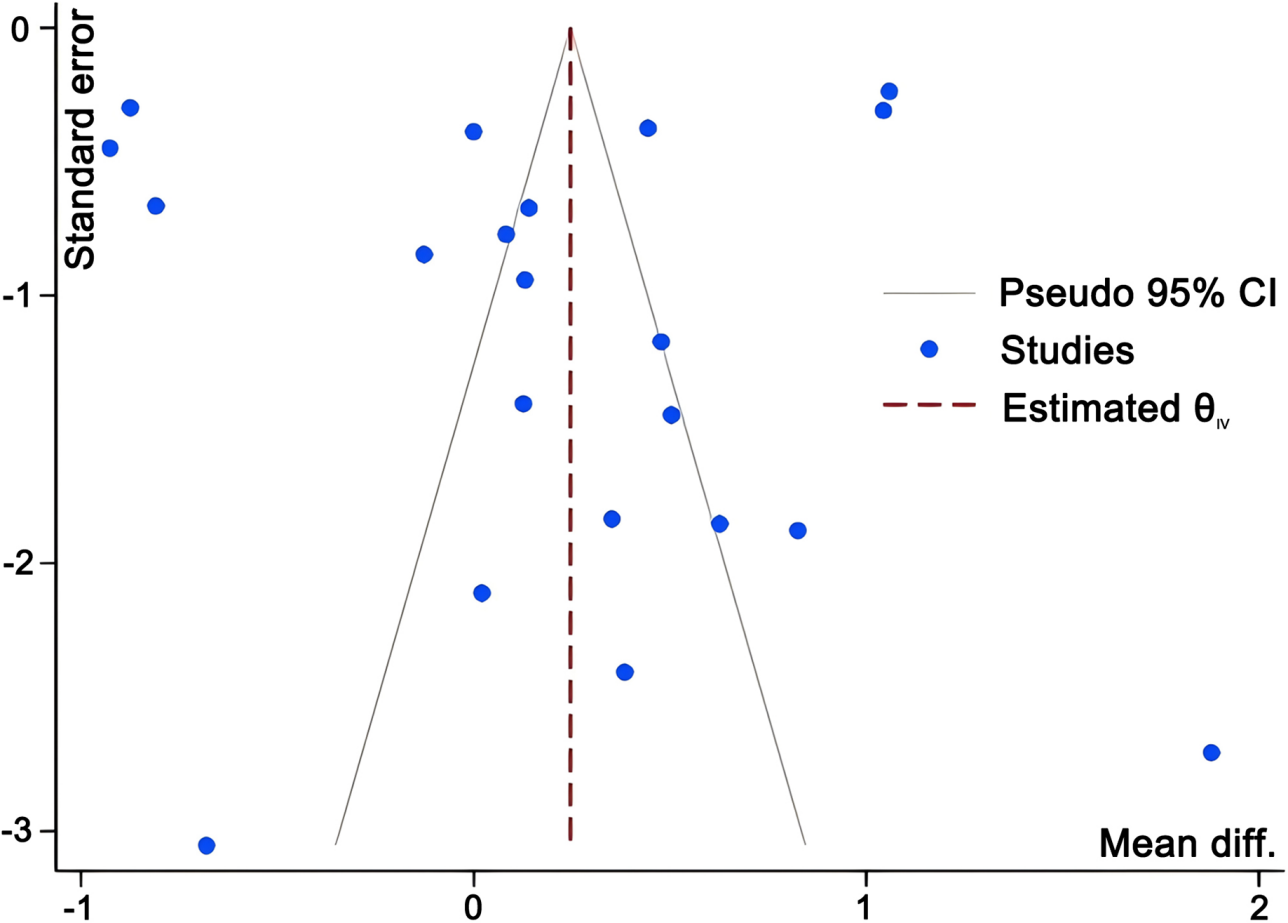
Funnel plot evaluating potential publication bias in studies investigating the effects of WRT on speed outcomes.

Funnel plots for strength and speed outcomes (Figs. 9 and 10) demonstrated relatively symmetrical distribution of effect sizes around the mean, with no obvious outliers. The trim-and-fill method showed that pooled effect sizes and 95% confidence intervals remained largely stable post-imputation. However, both Egger’s and Begg’s tests returned statistically significant results (*p* < 0.05) for strength and speed subgroups.

## Discussion

This study provides robust evidence that wearable resistance training (WRT) significantly enhances strength performance (Tables 1 and 2). Specifically, WRT demonstrated the most pronounced effect on lower-body explosive power (SMD = −0.60, *p* = 0.01), while its impact on maximal strength did not reach statistical significance (SMD = −0.09, *p* = 0.27). These findings demonstrate that WRT interventions confer the greatest benefits for jumping power and agility performance. The substantial heterogeneity observed for jumping power (I^2^ = high, Q(13) = 137.39, *p* = 0.01) suggested the presence of potential effect moderators, which led to subgroup analyses examining training load, intervention duration, and training frequency as key variables potentially influencing intervention effectiveness.

**Table 1:** Basic characteristics of the included studies (explosive strength).

Author	Participant characteristics	Intervention duration	Training frequency	Practice format	Load position	Load weight	Outcome indicators
Bosco et al. (1984)	Male, *n* = 6, age: 25.5 ± 3.24, TE: >7 y	3 weeks	7 per/week	DT	Trunk	13%	Squat jump
Bosco (1985)	Male, *n* = 5, age: 26.0 ± 2.2, TE: >7 y	3 weeks	7 per/week	DT	Trunk	11%	Squat jump, vertical jump
Bosco, Rusko & Hirvonen (1986)	Male/Female, *n* = 7, 22 ± 4.1, TE: 5–15 y	3 weeks	4 per/week	DT	Trunk	8%	Countermovement jump
Bustos et al. (2020)	Male, *n* = 15, 15–18, TE: >5 y	8 weeks	3 per/week	WU	Leg	1%	Squat jump, countermovement jump
Khlifa et al. (2010)	Male, *n* = 9, age: 23.61 ± 0.96, TE: 12.4 ± 3.5 y	10 weeks	3 per/week	ST	Trunk	11%	Squat jump, countermovement jump, five-repetition continuous jump
Markovic et al. (2013)	Male, *n* = 11, 23.7 ± 1.7	8 weeks	3 per/week	ST	Trunk	30%	Squat jump, countermovement jump
Marriner et al. (2017)	Male, *n* = 8, age: 23.2 ± 2.7, TE: 5.1–5.3	5 weeks	3 per/week	ST	Trunk	12%	Countermovement jump
Rey et al. (2017)	Male, *n* = 10, age: 23.7 ± 4.5, TE: 14.7 ± 4.1	6 weeks	2 per/week	SP	Trunk	18.9%	Countermovement jump
Sands et al. (1996)	Female, *n* = 5, age: 18–21	3 weeks	7 per/week	DT	Trunk	10%	Vertical jump
Scudamore et al. (2016)	Male, *n* = 9, age: 21 ± 2, TE: >1	3 weeks	3 per/week	DT	Trunk	11.2%	Countermovement jump
Simpson et al. (2020)	Female, *n* = 9, age: 21	3 weeks	4 per/week	DT	Trunk	8%	Countermovement jump
Del Vecchio et al. (2021)	Male, *n* = 10, age: 28.53 ± 1.87, TE: >2	6 weeks	2 per/week	CT	Leg	3.3%	Vertical jump
Ryan et al. (2025)	Female, *n* = 15, age: 15.96 ± 0.82 y, TE: 3–5 y	6 weeks	2 per/week	AT	Leg	2.5%	Vertical jump
Rantalainen, Ruotsalainen & Virmavirta (2012)	Male, *n* = 8, 32 ± 6	3 weeks	3 per/week	DT	Leg	5.6%	Countermovement jump

**Note:**

ST, Strength training; SPT, speed training; DT, daily training; WU, warm up; CT, combat training.

**Table 2:** Basic characteristics of the included studies (maximum strength).

Author	Participant characteristics	Intervention duration	Training frequency	Practice format	Load position	Load weight	Outcome indicators
Marriner et al. (2017)	Male, *n* = 8, age: 23.2 ± 2.7, TE: 5.1–5.3	5 weeks	3 per/week	ST	Trunk	12%	One-repetition maximum (1RM) power clean
Markovic et al. (2013)	Male, *n* = 11, 23.7 ± 1.7	8 weeks	3 per/week	ST	Trunk	30%	One-repetition maximum (1RM) squat
Scudamore et al. (2016)	Male, *n* = 9, age: 21 ± 2, TE: >1	3 weeks	3 per/week	DT	Trunk	11.2%	One-repetition maximum (1RM) power clean
Del Vecchio et al. (2021)	Male, *n* = 10, age: 28.53 ± 1.87, TE: >2	6 weeks	2 per/week	CT	Leg	3.3%	Five-repetition maximum (5RM) squat, five-repetition maximum (5RM) bench press

**Note:**

ST, Strength training; SPT, speed training; DT, daily training; WU, warm up; CT, combat training.

Training load, intervention duration, and training frequency were identified as potential key variables influencing intervention efficacy. To further elucidate these relationships, subgroup analyses were conducted, revealing that weekly training frequency and training load play crucial roles in determining outcomes. A training frequency of three sessions per week yielded optimal results for jump performance (SMD = 0.22, *p* = 0.038). Regarding training load, the application of 8–19% body weight resistance per session produced the most favorable effects on jump performance (SMD = −0.17, *p* = 0.032). WRT also demonstrated significant positive effects on agility (SMD = −0.44, *p* < 0.01). In contrast, the impact of WRT on speed did not achieve statistical significance (SMD = 0.25, *p* = 0.06), indicating that its effectiveness in enhancing speed performance remains equivocal and warrants further investigation.

1. Effects of WRT on strength performance

Previous research has demonstrated that enhancement of muscular strength performance requires improved neural system adaptability and recruitment efficiency, typically achieved through increased muscle firing frequency and motor unit recruitment. Markovic et al. (2013) reported that following 3 weeks of WRT intervention, the force-velocity curve exhibited a notable rightward shift (11%), accompanied by significant increases in power output during consecutive jumps. Research findings indicate that WRT can enhance maximal strength in both upper-body bench press (Del Vecchio et al., 2017) and lower-body squat exercises (Markovic et al., 2013). However, the magnitude of improvement in maximal strength is considerably less pronounced than that observed in lower-body explosive power (jump performance). This differential response may be attributed to WRT providing optimal stimuli for power development but insufficient mechanical tension for maximum strength adaptations. The enhancement of maximal strength follows the principle of supercompensation, wherein the application of high-intensity loads exceeding 85% of one-repetition maximum (1RM) constitutes a fundamental stimulus for eliciting maximal strength adaptations. Training intensity must reach a sufficient threshold to trigger the corresponding physiological adaptations necessary for meaningful strength gains (Folland & Williams, 2007). The limited efficacy of WRT for developing maximum strength is further confirmed by the scarcity of relevant literature, with only four studies examining maximum strength outcomes. Therefore, the subsequent discussion primarily focuses on the analytical examination of WRT effects on lower-limb explosive power performance.

Subgroup analysis results demonstrated that a training frequency of three sessions per week significantly enhanced jump performance (*p* < 0.05). The significant between-group difference (*p* = 0.04) compared to seven sessions per week, which paradoxically showed negative effects, indicates that training frequency is a critical moderating factor. This finding aligns with recovery physiology principles: the fundamental principle of power training lies in promoting neural system adaptations and enhancing muscular force output capacity (Gilbert & Lees, 2005). According to Selye’s General Adaptation Syndrome theory (Selye, 1936), the body undergoes three distinct phases following training stimuli: the Alarm Reaction Stage, the Resistance Stage, and the Exhaustion Stage. The effectiveness of rapid strength training largely depends on whether the central nervous system maintains optimal excitability. A training frequency of three sessions per week provides sufficient recovery windows for the body, facilitating the neuromuscular system’s adaptive enhancement processes and thereby more effectively improving explosive power performance. McLester, Bishop & Guilliams (2000) demonstrated that when total training volume was equated, a frequency of three sessions per week was superior to once-weekly training for strength and power development, as it enhances motor unit recruitment efficiency while reducing neural fatigue per training session. However, relatively few studies have directly investigated the optimal weekly frequency for WRT interventions. Training frequency does not operate in isolation but rather interacts with other factors, such as intensity, load volume, and exercise specificity, to influence performance outcomes. Further investigation is needed to clarify the dose-response relationship between training frequency and strength outcomes, as well as to identify the optimal frequency for maximizing performance adaptations across diverse athletic populations.

Regarding training load, the present study indicates that utilizing 8–19% of body weight as resistance during each training session effectively enhances jump performance (*p* < 0.05, Table 3). Specifically, Marriner et al. (2017) found that 5 weeks of explosive training with a 12% body mass load on the lower limbs and back led to significant improvements in jumping ability, explosive power, and one-repetition maximum (1RM) strength when compared to an unloaded control group. They suggested that implementing a 12% load maintains overall training intensity while reducing mechanical stress, thereby enabling athletes to focus on technical refinement without compromising movement quality due to excessive loading. Similarly, Scudamore et al. (2016) observed that 3 weeks of jump training with a trunk load of 11.2–16.1% body weight resulted in marked gains in jump performance, though variations in loading protocols had differing impacts on muscular power and jumping mechanics. In contrast, Bustos et al. (2020) conducted an 8-week intervention with national-level soccer players, comparing a WRT group (with calf loading at 0.58% of body mass) to a control group. The study found no significant improvements in countermovement jump (CMJ) performance in either group, suggesting that mismatched movement patterns or insufficient load magnitude and intensity in WRT may fail to elicit the neuromuscular adaptations required for vertical jump enhancement. Future research should consider movement-specific training protocols or appropriate Wearable Resistance load selection to facilitate improvements in jump performance (Ebben & Jensen, 2002).

**Table 3:** Subgroup analysis of the effects of wearable resistance training on explosive strength.

Subgroup			Studies (*n*)	Meta-analysis				
				SMD	(95% CI)	*P* ^within^	I^2^ (%)	*p* ^between^
Explosive strength	Intervention duration (weeks)	1–3	6	−0.11	[−0.31 to 0.10]	0.309	74.14	0.08
		6–8	4	0.13	[−0.03 to 0.29]	0.114	47.73	
	Intervention frequency (times/week)	2	3	0.03	[−0.17 to 0.22]	0.799	68.75	0.04*
		3	3	−0.47	[−0.92 to −0.02]	0.038	49.00	
		4	2	−0.11	[−0.44 to 0.23]	0.528	85.79	
		7	2	0.22	[0.01 to 0.42]	0.041	63.52	
	Wearing load (body weight%)	1–6	4	0.18	[0.01 to 0.34]	0.099	63.68	0.01*
		8–19	6	−0.17	[−0.37 to 0.03]	0.032	59.40	
	Wearing position	Trunk	7	−0.07	[−0.25 to 0.12]	0.478	71.19	0.12
		Leg	3	0.13	[−0.04 to 0.31]	0.128	64.37	
	Participant age	Adults	7	−0.01	[−0.19 to 0.17]	0.926	57.32	0.46
		Youth	3	0.09	[−0.09 to 0.26]	0.338	90.56	
	Participant population	Athletes	8	0.03	[−0.10 to 0.17]	0.636	71.97	0.81
		Non-athletes	2	0.08	[−0.24 to 0.39]	0.641	81.85	

**Note:**

SMD, Standardized mean difference; CI, confidence interval. Bold values with asterisks (*) indicate statistical significance at *p* < 0.05.

2. Effects of WRT on speed performance

Meta-analysis results indicated that WRT demonstrated a trend toward improving speed performance (Table 4), although this effect did not reach statistical significance (*p* = 0.06). These findings align with those reported by Macadam, Simperingham & Cronin (2019). As a fundamental component of human locomotion, sprint capability is widely recognized as a critical determinant of athletic success across numerous sports disciplines (Macadam, Simperingham & Cronin, 2019; Hunter, Marshall & Mcnair, 2004). Key factors influencing sprint performance include the coordination between stride frequency and stride length, the capacity to generate substantial force rapidly, and the efficiency of force application during ground contact phases (Cross et al., 2018).

**Table 4:** Basic characteristics of the included studies (speed).

Author	Participant characteristics	Intervention duration	Training frequency	Practice format	Load position	Load weight	Outcome indicators
Barr et al. (2015)	Male, *n* = 8, age: 22.21 ± 2.36 y	8 days	7 per/week	ST, SPT	Trunk	12%	10 m sprint, 40 m sprint
Bustos et al. (2020)	Male, *n* = 15, 15–18, TE: >5 y	8 weeks	3 per/week	WU	Trunk	1%	10 m sprint, 20 m sprint
Clark et al. (2010)	Male, *n* = 7, age: 19.7–19.9 y	7 weeks	2 per/week	SPT	Trunk	18.5%	18.3–54.9 m sprint
Feser et al. (2021a)	Male, *n* = 12, age: 23.51 ± 3.07, TE: >1 y	6 weeks	2 per/week	SPT	Leg	1%	10 m sprint, 20 m sprint, 5 m sprint, 30 m sprint
Feser et al. (2021b)	Male, *n* = 11, age: 16.47 ± 0.49, TE: >1 y	9 weeks	2 per/week	SPT	Leg	1%	10 m sprint, 20 m sprint, 5 m sprint, 30 m sprint
Pajić et al. (2011)	Male, *n* = 6, age: 20.47 ± 1.59	6 weeks	3 per/week	ST	Leg	2.5%	50 m sprint
Rey et al. (2017)	Male, *n* = 10, age: 23.7 ± 4.5, TE: 14.7 ± 4.1 y	6 weeks	2 per/week	SPT	Trunk	18.9%	10 m sprint, 20 m sprint
Rantalainen, Ruotsalainen & Virmavirta (2012)	Male, *n* = 8, 32 ± 6	3 weeks	3 per/week	DT	Leg	5.6%	10 m sprint
Scudamore et al. (2016)	Male, *n* = 9, age: 21 ± 2, TE: >1 y	3 weeks	4 per/week	DT	Trunk	11.2%	36.58 m sprint
Simpson et al. (2020)	Female, *n* = 9, age: 21 y	3 weeks	4 per/week	DT	Trunk	8%	25 m sprint
Ryan et al. (2025)	Female, *n* = 15, age: 15.96 ± 0.82 y, TE: 3–5 y	6 weeks	2 per/week	AT	Leg	2.5%	10 m sprint, 30 m sprint

**Note:**

ST, Strength training; SPT, speed training; DT, daily training; WU, warm up; AT, agility training.

In WRT, external loads for improving sprint performance are usually applied to the trunk or lower limbs. Trunk loading typically ranges from 9% to 18.9% of body weight, while lower-limb loading often employs “micro-loads” between 1% and 5.6% of BM. The efficacy of WRT in enhancing speed performance remains contentious. Some research suggests that these resistance methods are designed to promote neuromuscular coordination and control, thereby enhancing the responsiveness and adaptability of the neuromuscular system (Scudamore et al., 2016). Additionally, WRT encourages the engagement of additional muscle fibers, thereby increasing both muscular strength and explosive power essential components for rapid acceleration. Empirical studies have demonstrated that WRT affects sprint-related kinematic variables, including ground contact time, stride length, stride frequency, and flight time (Gleadhill et al., 2021; Macadam, Cronin & Feser, 2022; Li et al., 2021). However, previous studies pointed out that while WRT may assist in maintaining or slightly improving sprint performance, its efficacy depends on several critical factors, including load magnitude, placement, progression strategy, and individual variability. Improper application may result in uneven loading or extreme fatigue, potentially undermining training outcomes. Feser et al. (2021a, 2021b, 2021c, 2023) conducted two relevant experiments. In the first study (Feser et al., 2021a), researchers implemented a 6-week intervention program with collegiate rugby players, employing bilateral calf loading at 1% body weight combined with rugby-specific running and jumping drills. Results revealed non-significant declining trends across all sprint metrics at 5, 10, 20, and 30 m distances. In a subsequent investigation (Feser et al., 2021b), the same research team shifted their focus to high school rugby players, extending the intervention duration to 9 weeks. The findings indicated improvements in short-distance sprint performance (10, 20, 30 m), although these changes did not reach statistical significance (*p* > 0.05). The greater adjusted mean theoretical maximal velocity scores (*p* > 0.05; ES = 0.30) found for the WR group compared to the control group at post-intervention may suggest that WR amplifies the nuances of the training protocol itself. Moreover, baseline strength, conditioning level, and adaptability further influence individual responses to WRT. Bosco (1985), Bosco et al. (1984) similarly contended that 3 weeks of WRT is inadequate to bring about significant mechanical adjustments in the leg muscles. Given that the participants were elite athletes already performing at their peak, achieving further enhancements in sprint capacity *via* WRT alone was considered improbable. Finally, Macadam, Simperingham & Cronin (2019) highlighted the persistent ambiguity surrounding the effectiveness of weighted vests in improving short-distance sprint performance. Similar to traditional resistance training methods, WRT may necessitate extended intervention durations to elicit meaningful performance gains (Rantalainen, Ruotsalainen & Virmavirta, 2012).

In summary, although WRT demonstrated positive trends for speed performance, these effects did not achieve statistical significance. Consequently, subgroup analyses examining factors such as loading placement were not conducted. Future high-quality research is warranted to elucidate the impact of different loading positions and magnitudes on speed performance enhancement.

3. Effects of WRT on agility performance

The present study demonstrates that WRT significantly enhances agility performance (Table 5). Agility, defined as the capacity to respond to external stimuli and rapidly change direction, velocity, or movement patterns (Silva et al., 2023), represents a critical athletic attribute. Effective strategies for enhancing agility include short sprints, deceleration drills, change-of-direction (COD) tasks, complex strength exercises, and sport-specific plyometric training (Chen et al., 2025; Negra et al., 2020). WRT provides a flexible overload stimulus that can be seamlessly incorporated into warm-up routines, conditioning protocols, or simulated gameplay. A crucial aspect of agility is braking ability, and WRT has been demonstrated to increase vertical load, thereby enhancing ground reaction force and braking capacity. Rantalainen, Ruotsalainen & Virmavirta (2012) observed a 2.1% improvement in agility, assessed through the figure-of-8 test, following a 3-week intervention where eight healthy male participants wore weighted vests during their daily activities. More recently, Ryan et al. (2025) reported that 6 weeks of calf-loaded WRT in adolescent female field hockey players integrated calf-loaded WRT into their 6-week warm-up training, resulted in significant reductions in 5-0-5 test times (7.14%) and 180° turn times (9.29%) (*p* < 0.05) (Ryan et al., 2025). Li et al. (2021) found that trunk loading at 5% of body mass acutely enhanced change-of-direction performance in soccer players, Bustos et al. (2020) verified significant COD improvements following 8 weeks of calf-loaded WRT. Elite-level athletes with exceptional multiplanar agility and cutting proficiency consistently achieve locomotor dominance under temporal pressure and high-stakes game scenarios, thereby optimizing tactical execution and technical precision (Istvan Rydsa & van den Tillaar, 2020). Currently, evidence supporting WRT application strategies aimed at optimizing change-of-direction performance remains extremely limited, precluding the establishment of consensus guidelines. Future research should further investigate the effects of different load placements, loading intensities, and exercise modalities within WRT on change of direction (COD) performance enhancement.

**Table 5:** Basic characteristics of the included studies (agility).

Author	Participant characteristics	Intervention duration	Training frequency	Practice format	Load position	Load weight	Outcome indicators
Bustos et al. (2020)	Male, *n* = 15, 15–18, TE: >5 y	8 weeks	3 per/week	WU	Trunk	1%	20 m shuttle run
Rantalainen, Ruotsalainen & Virmavirta (2012)	Male, *n* = 8, 32 ± 6	3 weeks	3 per/week	DT	Leg	5.6%	Figure-of-8 run
Ryan et al. (2025)	Female, *n* = 15, age: 15.96 ± 0.82, TE: 3–5	6 weeks	2 per/week	AT	Leg	2.5%	5-0-5 test, 180° change-of-direction test
Scudamore et al. (2016)	Male, *n* = 9, age: 21 ± 2, TE: >1	3 weeks	4 per/week	DT	Trunk	11.2%	137.2 m shuttle run
Simpson et al. (2020)	Female, *n* = 9, age: 21	3 weeks	4 per/week	DT	Trunk	8%	T-test

**Note:**

ST, Strength training; SPT, speed training; DT, daily training; WU, warm up; AT, agility training.

### Limitations and future directions

Several limitations should be considered when interpreting these findings. First, the included studies demonstrated substantial heterogeneity in key training parameters, including external load (ranging from 1% to 30% of body weight), intervention duration, and training frequency. Furthermore, the participants’ training histories were highly diverse, making it challenging to determine a universally optimal loading prescription suitable for regular athletic practice. Other factors examined (placement location, age, participant population type) showed no significant moderating effects, though this may reflect limited statistical power rather than true absence of effects. Additionally, due to the limited number of high-quality investigations, this review could not definitively determine the moderating effects of loading placement (*e.g*., trunk *vs*. lower limbs) on speed performance. Future research should systematically investigate optimal load magnitudes, placement configurations, and progressive overload protocols, to optimize performance enhancement across diverse athletic populations to establish evidence-based guidelines for WRT implementation.

## Conclusions

WRT presents a practical and effective strategy for integrating resistance with technical training while maintaining movement precision. The results of this review indicate that WRT is most beneficial for enhancing muscular strength and agility, although significant improvements in sprint performance have yet to be demonstrated. The enhancement of strength performance through WRT is primarily manifested in lower-limb explosive power. For developing jumping capacity, a training protocol of three sessions per week with loading intensities ranging from 8% to 19% of body weight is recommended for WRT implementation. These training parameters were particularly effective in enhancing jump-related performance metrics. However, the impact of load placement (*e.g*., trunk *vs*. lower limbs) on sprint performance remains uncertain. Future research should explore the combined effects of load configuration, placement, and progression strategies to identify the most effective approaches for optimizing sprint-related adaptations. Practically, coaches and practitioners are encouraged to consider WRT as a complementary component in organized strength and conditioning programs. Future research should prioritize investigating the potential effects of WRT load magnitude, direction, and placement on athletic performance to establish optimal guidelines for implementing WRT in athletic populations.

## Supplemental Information

10.7717/peerj.20519/supp-1 Supplemental Information 1 PRISMA Checklist.

10.7717/peerj.20519/supp-2 Supplemental Information 2 Search strategy.

## Additional Information and Declarations

### Data Availability

The following information was supplied regarding data availability:

This is a systematic review/meta-analysis.
