# Supplementary material for: The impact of wearable resistance training on strength, speed, and agility: a systematic review and meta-analysis
Source: PeerJ. 2026 Jan 2;14:e20519. doi: 10.7717/peerj.20519 (PMC12767494; doi:10.7717/peerj.20519)
Supplement: Supplemental Information 1 [file peerj-14-20519-s001.docx]

PRISMA 2020 Checklist

For: The Impact of Wearable Resistance Training on Strength, Speed, and Agility: A Systematic Review and Meta-analysis

| Item | Location | Remarks | Note |
| --- | --- | --- | --- |
| 1 | Title | Title page, line 1 | Indicate that it is a systematic review or Meta - analysis |
| 2 | Abstract | Lines 14 - 39, page 1 | Structured abstract covering background, objectives, methods, results, and conclusions |
| 3 | Introduction | From line 40, page 1 to line 65, page 2 | Elaborate on research background and objectives |
| 4 | Methods - Search Strategy | Lines 73 - 85, page 2 | Specify all databases and search time |
| 5 | Methods - Inclusion and Exclusion Criteria | Lines 86 - 116, page 2 | Clearly define inclusion and exclusion criteria |
| 6 | Methods - Data Extraction | Lines 117 - 134, page 3 | Clarify what data to extract and who will conduct the extraction |
| 7 | Methods - Bias Assessment | Lines 135 - 144, page 3 | Explain the method and tool for bias assessment |
| 8 | Methods - Statistical Analysis | Lines 145 - 168, page 3 | Clarify data synthesis method (e.g., SMD, OR, etc.) |
| 9 | Results - Literature Screening Process | Lines 171 - 177, page 4 | Explain the inclusion process with Figure 1 |
| 10 | Results - Study Characteristics | Lines 178 - 184, page 4 | Clarify study count, sample size, intervention characteristics |
| 11 | Results - Risk of Bias | Lines 185 - 193, page 4 | Explain study quality with Figure 2 |
| 12 | Results - Effect Synthesis | Lines 194 - 238, pages 4 - 5 | Explain synthesized results of each index with Figure 5 - 7 |
| 13 | Results - Subgroup and Regression | Lines 223 - 273, page 5 | Analyze heterogeneity sources with Table 4 - 5 |
| 14 | Results - Publication Bias | Lines 274 - 290, page 5 | Explain with Figure 8 - 9 and statistical test results |
| 15 | Discussion | Lines 292 - 440, pages 6 - 8 | Explain main results, mechanisms, and significance |
| 16 | Limitations | Lines 441 - 458, page 8 | Potential bias and study limitations |
| 17 | Conclusion | Lines 460 - 477, page 8 | Clearly summarize study results |
| 18 | Other Information - Data Availability | Line 479, page 8 | Clarify how to access data |
| 19 | Other Information - Ethics Statement | Line 482, page 8 | Note ethics approval status |
| 20 | Other Information - Author Contributions, Funding, and Conflicts of Interest | Lines 485 - 494, page 8 | List author roles, funding details |
