# Supplementary material for: The impact of wearable resistance training on strength, speed, and agility: a systematic review and meta-analysis
Source: PeerJ. 2026 Jan 2;14:e20519. doi: 10.7717/peerj.20519 (PMC12767494; doi:10.7717/peerj.20519)
Supplement: Supplemental Information 2 [file peerj-14-20519-s002.docx]

| PubMed search strategy | ("Resistance Training"[Mesh] OR "resistance training" OR "weight training" OR "strength training" OR "wearable resistance" OR "loaded movement training" OR "weight-bearing exercise")  AND  ("Muscle Strength"[Mesh] OR "strength" OR "muscular strength" OR "power" OR "force production" OR "Athletic Performance"[Mesh] OR "speed" OR "velocity" OR "sprint performance" OR "running speed" OR "Physical Functional Performance"[Mesh] OR "agility" OR "change of direction ability" OR "quickness" OR "motor agility") |
| --- | --- |
| Web of Science search strategy | TS=("resistance training" OR "weight training" OR "strength training" OR "wearable resistance" OR "loaded movement training" OR "weight-bearing exercise")  AND  TS=("strength" OR "muscular strength" OR "power" OR "force production" OR "speed" OR "velocity" OR "sprint performance" OR "running speed" OR "agility" OR "change of direction ability" OR "quickness" OR "motor agility") |
| EBSCO search strategy | (DE "RESISTANCE training" OR "resistance training" OR "weight training" OR "strength training" OR "wearable resistance" OR "loaded movement training" OR "weight-bearing exercise")  AND  (DE "MUSCLE strength" OR "strength" OR "muscular strength" OR "power" OR "force production" OR DE "ATHLETIC ability" OR "speed" OR "velocity" OR "sprint performance" OR "running speed" OR "agility" OR "change of direction ability" OR "quickness" OR "motor agility") |
